# Characterization of the wave phenomenon of flash-induced chlorophyll fluorescence in *Chlamydomonas reinhardtii*

**DOI:** 10.1007/s11120-022-00900-3

**Published:** 2022-02-15

**Authors:** Priyanka Pradeep Patil, Sabit Mohammad Aslam, Imre Vass, Milán Szabó

**Affiliations:** 1 Biological Research Centre, Institute of Plant Biology, Eötvös Loránd Research Network (ELKH), Szeged, Hungary; 2 Doctoral School of Biology, University of Szeged, Szeged, Hungary; 3 Climate Change Cluster, University of Technology Sydney, Ultimo, Australia

**Keywords:** Chlorophyll fluorescence, Photosystem II, Microalgae, Plastoquinone pool, Linear electron transport, Cyclic electron transport

## Abstract

Flash-induced chlorophyll fluorescence relaxation is a powerful tool to monitor the reoxidation reactions of the reduced primary quinone acceptor, Q_A_^−^ by Q_B_ and the plastoquinone (PQ) pool, as well as the charge recombination reactions between the donor and acceptor side components of Photosystem II (PSII). Under certain conditions, when the PQ pool is highly reduced (e.g. in microaerobic conditions), a wave phenomenon appears in the fluorescence relaxation kinetics, which reflects the transient reoxidation and re-reduction of Q_A_^−^ by various electron transfer processes, which in cyanobacteria is mediated by NAD(P)H dehydrogenase (NDH-1). The wave phenomenon was also observed and assigned to the operation of type 2 NAD(P)H dehydrogenase (NDH-2) in the green alga *Chlamydomonas reinhardtii* under hydrogen-producing conditions, which required a long incubation of algae under sulphur deprivation (Krishna et al. J Exp Bot 70 (21):6321–6336, 2019). However, the conditions that induce the wave remained largely uncharacterized so far in microalgae. In this work, we investigated the wave phenomenon in *Chlamydomonas reinhardtii* under conditions that lead to a decrease of PSII activity by applying hydroxylamine treatment, which impacts the donor side of PSII in combination with a strongly reducing environment of the PQ pool (microaerobic conditions). A similar wave phenomenon could be induced by photoinhibitory conditions (illumination with strong light in the presence of the protein synthesis inhibitor lincomycin). These results indicate that the fluorescence wave phenomenon is activated in green algae when the PSII activity decreases relative to Photosystem I (PS I) activity and the PQ pool is strongly reduced. Therefore, the fluorescence wave could be used as a sensitive indicator of altered intersystem electron transfer processes, e.g. under stress conditions.

## Introduction

Photosynthesis is a series of redox reactions, in which several electron transport processes operate to provide the energetic balance of light harvesting. Besides linear electron flow, which ensures the basic functions of photosynthetic productivity and carbon fixation, alternative electron transport pathways operate, such as the cyclic electron transport, which play a role in fine-tuning photosynthesis and balancing the ATP/NADPH ratio under stress conditions (Kramer and Evans 2011). Cyclic electron flow (CEF) involves transfer of electrons from ferredoxin or NADPH back to the PQ pool, which is then transferred to cyt b_6_/f and again to PSI without the net production of NADPH. However, ATP production and proton gradient formation is maintained during CEF. There are two main pathways of CEF, the antimycin-sensitive pathway (involving ferredoxin PQ oxidoreductase) and the antimycin-insensitive pathway, involving NAD(P)H dehydrogenase (NDH-1 or NDH-2) (Hosler and Yocum 1987; Peltier et al. 2016). In *Chlamydomonas*, both pathways were shown to be functional (Desplats et al. 2009; Alric 2014); however, the NDH-2 pathway seems to be the main route of non-photochemical PQ pool reduction (Jans et al. 2008; Desplats et al. 2009; Mignolet et al. 2012; Baltz et al. 2014).

Flash-induced chlorophyll fluorescence relaxation has proved to be a useful method to study the kinetics of photosynthetic electron transport, which is related to the changes in the redox state of the Q_A_ primary quinone electron acceptor of Photosystem II (PSII) (Crofts and Wraight 1983; Robinson and Crofts 1983; Vass et al. , 1992, 1999). Single turnover flash illumination results in electron transfer from the water splitting complex to Q_A_ and the formation of Q_A_^−^ leads to a concomitant fluorescence increase. The flash-induced fluorescence then decreases monotonously reflecting three discernible phases of Q_A_^−^ reoxidation, (i) fast phase, electron transfer to Q_B_ which occurs in 300–500 μs, (ii) middle phase, reoxidation by PQ that binds to the Q_B_ site after the flash, which occurs in 5–15 ms, (iii) slow phase, occurring in 10–20 s, is due to Q_A_^−^ reoxidation via charge recombination with the oxidized S_2_ (or S_3_), which occurs from the Q_A_Q_B_^−^ state, which is in charge equilibrium with Q_A_^−^Q_B_ (Vass et al. 1999; Deák et al. 2014).

Earlier it was shown that *Synechocystis* cells incubated under microaerobic conditions exhibit a wave phenomenon in the fluorescence relaxation kinetics, which comprises a dip of 30–50 ms and a subsequent rise in a fluorescence with a local maximum at ~ 1 s, after which the fluorescence relaxes to the initial level (Deák et al. 2014). Microaerobic conditions lead to a highly reduced plastoquinone pool due to the impaired oxidation of the reduced PQ, PQH_2_, because the terminal oxidase (PTOX) is not functional, due to the lack of the terminal electron acceptor O_2_ (Houille-Vernes et al. 2011; Johnson and Alric 2013; Deák et al. 2014; Ermakova et al. 2016). The dip is related to the transient oxidation of the highly reduced PQ pool by the linear electron transport towards Photosystem I, and the following rise and the bump are related to subsequent re-reduction of plastoquinone pool, mediated by the type I NAD(P)H dehydrogenase (NDH-1) complex (Deák et al. 2014). In contrast, the wave phenomenon was not observed in *Chlamydomonas reinhardtii* under microaerobic conditions induced using glucose oxidase, catalase and glucose (Krishna et al. 2019), or achieved by algal–bacterial co-cultivation (Lakatos et al. 2014). On the other hand, the fluorescence wave appeared in sulphur-starved cells that downregulated photosynthetic O_2_ production, which resulted in the establishment of microaerobic conditions favoring H_2_ production (Antal et al. 2019; Krishna et al. 2019). In all cases, the fluorescence decay pattern was modified, typically slower fast and middle phases were observed, which is related to the elevated reduction level of the PQ pool, an important regulatory point of redox poise and intersystem electron transfer processes (Alric 2015). A significant wave phenomenon was also detected in the red morphotype of the green alga *Acetabularia acetabulum,* a prey of the photosynthetic sea slug *Elysia timida* under aerobic conditions (Havurinne and Tyystjärvi 2020). Despite these recent characterizations of the chlorophyll fluorescence relaxation kinetics in some algae and cyanobacteria, the chlorophyll fluorescence signature representing Q_A_^−^ reoxidation processes by the intersystem electron transfer from PSII to PSI, and the influence of stromal reductants and cyclic electron flow on the flash-induced fluorescence relaxation kinetics remain largely unknown in microalgae. The wave phenomenon in *Chlamydomonas* was found only under hydrogen-producing microaerobic conditions, when PSII activity is partly inhibited (Krishna et al. 2019). This observation raises the possibility that the PSII:PSI ratio, which have a strong influence on the redox state of the PQ pool following a flash (and also the spectrum of the light should be taken into account in this process, Mattila et al. 2020) can play an important role in the appearance of the fluorescence wave phenomenon.

In the present work we applied microaerobic conditions in combination with chemical or light treatments to inhibit PSII activity and thereby change the ratio of active PSII and PSI centers to reach a better understanding of the conditions which lead to the characteristic wave phenomenon of the flash-induced Chl fluorescence signal. The role of electron transport routes in the formation of the wave phenomenon was also investigated using specific inhibitors of the antimycin dependent and independent cyclic electron flow pathways. Our data show that in *Chlamydomonas reinhardtii*, and possibly in other green algae, the fluorescence wave can be observed only if the ratio of PSII and PSI activity decreases to sufficiently low level due partial inhibition of PSII. Therefore, the fluorescence wave is a sensitive indicator of altered intersystem electron transfer processes, e.g. under stress conditions.

## Materials and methods

### Algal cultures

*Chlamydomonas reinhardtii* CC-124 was grown in TAP medium at 24 °C, with continuous shaking at 56 μmol photons m^−2^ s^−1^ white light, 12 h:12 h light:dark cycle. Cells were harvested in early exponential phase (OD_720 nm_ of 0.2–0.3) and approx. 1 h after the onset of the daily light cycle, then centrifuged at 6500 g for 5 min and resuspended into fresh TAP medium to set Chl *a* content of 5 µg/ml. Chl content was determined spectrophotometrically using acetone:DMSO extraction according to (Shoaf and Lium 1976).

### Flash-induced chlorophyll fluorescence relaxation kinetics

Flash-induced chlorophyll fluorescence yield was measured using a double-modulation fluorometer (FL-3000, Photon System Instruments, Brno) (Trtilek et al. 1997). A 2 ml sample was placed in a cuvette with 1 cm pathlength and was continuously stirred with a small magnetic stirrer bar in the dark. Four measuring flashes (8 μs, separated with 200 μs intervals, wavelength of 620 nm) were applied to determine minimum fluorescence in the dark (*F*_0_), after which a single turnover saturating actinic flash (30 μs, wavelength of 639 nm) was given to induce the formation of Q_A_^−^, which resulted in the rise of fluorescence intensity. It has to be noted that the 30 μs long actinic flash can be considered as a single turnover flash for PSII but not for PSI, however this did not influence the findings in the current study. It is also important to emphasize that the actinic flash provided in the current experiments is not sufficient to reach maximal fluorescence (which is defined recently as light-adapted charge-separated state of PSII, Sipka et al. 2021), rather this is the maximal fluorescence recorded after the single turnover actinic flash, which we specifically denote as F_m(ST)_. The fluorescence decay resulting from reoxidation of Q_A_^−^ was measured by applying measuring flashes in the time range from 150 to 100 s on a logarithmic time scale (see also Patil et al. 2020). Variable fluorescence (*F*_v_) was calculated as *F*_v_ = *F*_m(ST)_ − *F*_0_, where *F*_m(ST)_ denotes maximal fluorescence recorded after the single turnover flash.

### Experimental procedure

The control samples (no inhibitor, aerobic conditions) were measured after dark adaptation for 3 min. In order to achieve highly reduced PQ pool by O_2_ depletion (which inhibits PQH_2_ oxidation by plastid terminal oxidase), the microaerobic condition was applied by the addition of 10 mM glucose, 7 U ml^−1^ glucose oxidase and 60 U ml^−1^ catalase, incubated in the dark for 15 min, after which the fluorescence decay curves were measured in the microaerobic state. In order to test the effect of hydroxylamine (NH_2_OH, abbreviated as ‘HA’ thereafter), parallel measurements were done, in which the samples were first incubated for 3 min with 1 mM HA, fluorescence curves were recorded, and subsequently the samples were subjected to microaerobic conditions in the presence of HA and fluorescence decay curves were recorded (‘HA + microaerobic conditions’).

For photoinhibition treatments 2 ml samples in a 1 cm cuvette were illuminated with 2000 μmol photons m^−2^ s^−1^ white light (Schott KL 1500, Heinz Walz GmbH, Effeltrich, Germany) for 15 min, in the presence of 1 mM lincomycin, after which samples were kept aerobic or microaerobic for 15 min and flash fluorescence was recorded.

For the treatments with CEF inhibitors, the above procedure for HA + microaerobic incubation was applied in the presence or absence of 10 μM antimycin A or 400 μM polymyxin B. These inhibitors were added to the cell suspensions before the application of hydroxylamine and microaerobic treatment.

Experiments were performed in triplicates, and averaged fluorescence traces with standard deviation were plotted using OriginPro (OriginLab Corp. Northampton, MA, USA).

### Statistical analysis

Statistical analysis was performed using OriginPro (OriginLab Corporation, Northampton, MA, USA). One-way analysis of variance (ANOVA) was performed on independent samples to detect statistically significant differences between treatments for which Tukey’s post hoc multiple comparison tests (α = 0.05) were applied. Normality tests were performed using the Kolmogorov–Smirnov method and the homogeneity of variance test was performed using Levene’s method.

## Results

### Partial inhibition of PSII activity by hydroxylamine treatment leads to a fluorescence wave in *Chlamydomonas* under microaerobic conditions

**Fig. 1:**
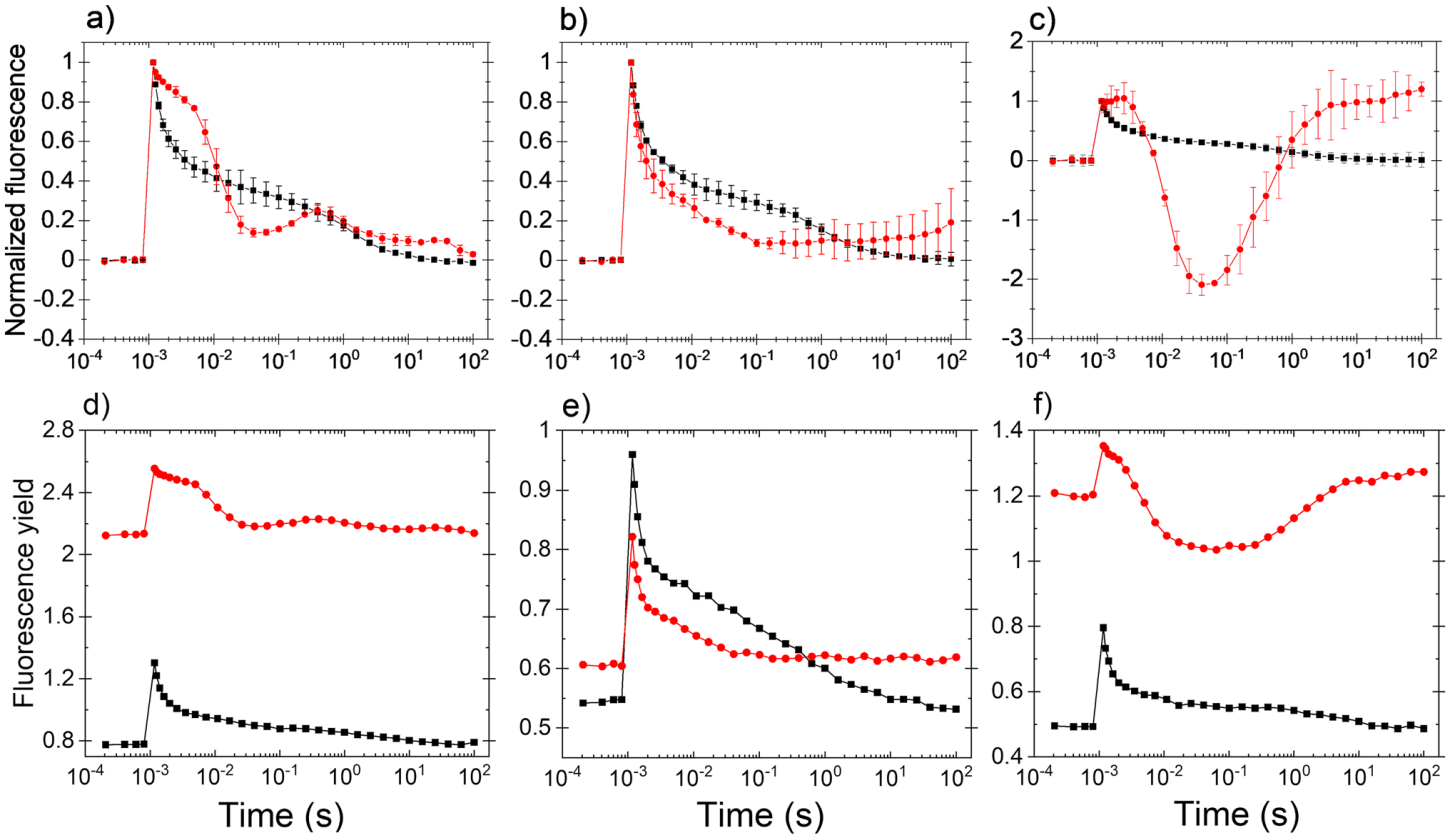
Effect of HA treatment on flash-induced Chl fluorescence relaxation in *C. reinhardtii*. **a** Control cells without inhibitor under aerobic (black) and microaerobic (red) conditions. **b** HA-treated cells under aerobic conditions (red). **c** HA-treated cells under microaerobic conditions (red). The black traces in panels **b** and **c** show the aerobic untreated control as in panel **a**. Curves shown in panels **a**, **b** and **c** were double normalized (*F*_0_ to 0 and *F*_m(ST)_, which represents maximal fluorescence after the flash to 1) to achieve the same initial amplitudes. Panels **d**, **e** and **f** show the original traces of the respective conditions without normalization

Upon microaerobic treatment, a strong increase in *F*_0_ was observed in *C. reinhardtii*, and concomitantly the fast phase of fluorescence decay slowed down. Although a complete wave that is typical for *Synechocystis* was not detected, a wave-like phenomenon with a dip at 0.06–0.1 s and a bump at 3–5 s appeared under microaerobic conditions (Fig. 1a). The elevated F_0_ before the flash under microaerobic conditions, observed together with slower fluorescence decay in the fast phase and an elevated middle phase indicate the presence of a strongly reduced PQ pool (Fig. 1d).

Since the proper balance of Photosystem I (PSI) and PSII electron transport activities is critical for induction of the wave phenomenon (Deák et al. 2014), we modulated this balance by inhibiting PSII activity at its donor side by applying hydroxylamine (HA) treatment, under aerobic and microaerobic conditions. HA releases the Mn cluster from PSII and therefore decreases PSII activity without affecting PSI activity (Cheniae and Martin 1971). In the presence of HA PSII activity was largely abolished, indicated by a significant, 40–50% decrease in the amplitude of variable fluorescence (F_v_), and a small, statistically not significant increase in minimal fluorescence (F_0_) (Fig. 1e and see Fig. 3a below). However, the linear electron flow downstream of Q_A_ was undisturbed, as the fast phase of Chl relaxation was similar in non-treated vs. HA-treated cells (Fig. 1b). In the middle phase of decay, in the HA-treated cells the fluorescence was even below the fluorescence level of non-treated cells, indicating the efficient intersystem electron transfer from Q_A_^−^ to Q_B_ and the PQ pool and an efficient reoxidation of PQH_2_ by PSI. A dip in Chl fluorescence decay was observed with a minimum at around 0.1 s, followed by a slow re-reduction (Chl fluorescence rise) in 1–10 s, but this did not manifest in a bump in HA-treated cells. The lack of slow phase indicates the lack of charge recombination between Q_B_ and S_2_ state, most probably because of the dysfunctional PSII donor side (Vass et al. 1999)*.*

When the cells were incubated with HA and then microaerobic treatment was applied, F_0_ was strongly elevated as compared to the non-treated cells (Fig. 1f). After the flash, the fast phase of the decay was remarkably slow, but the fluorescence strongly decreased thereafter and a large dip phase was observed at around 0.05–0.06 s. Then the fluorescence increased and peaked at 3–4 s and the slow phase of the decay was completely missing (Fig. 1c).

### Partial inhibition of PSII activity by high light treatment leads to a fluorescence wave in *Chlamydomonas* under anaerobic conditions

**Fig. 2:**
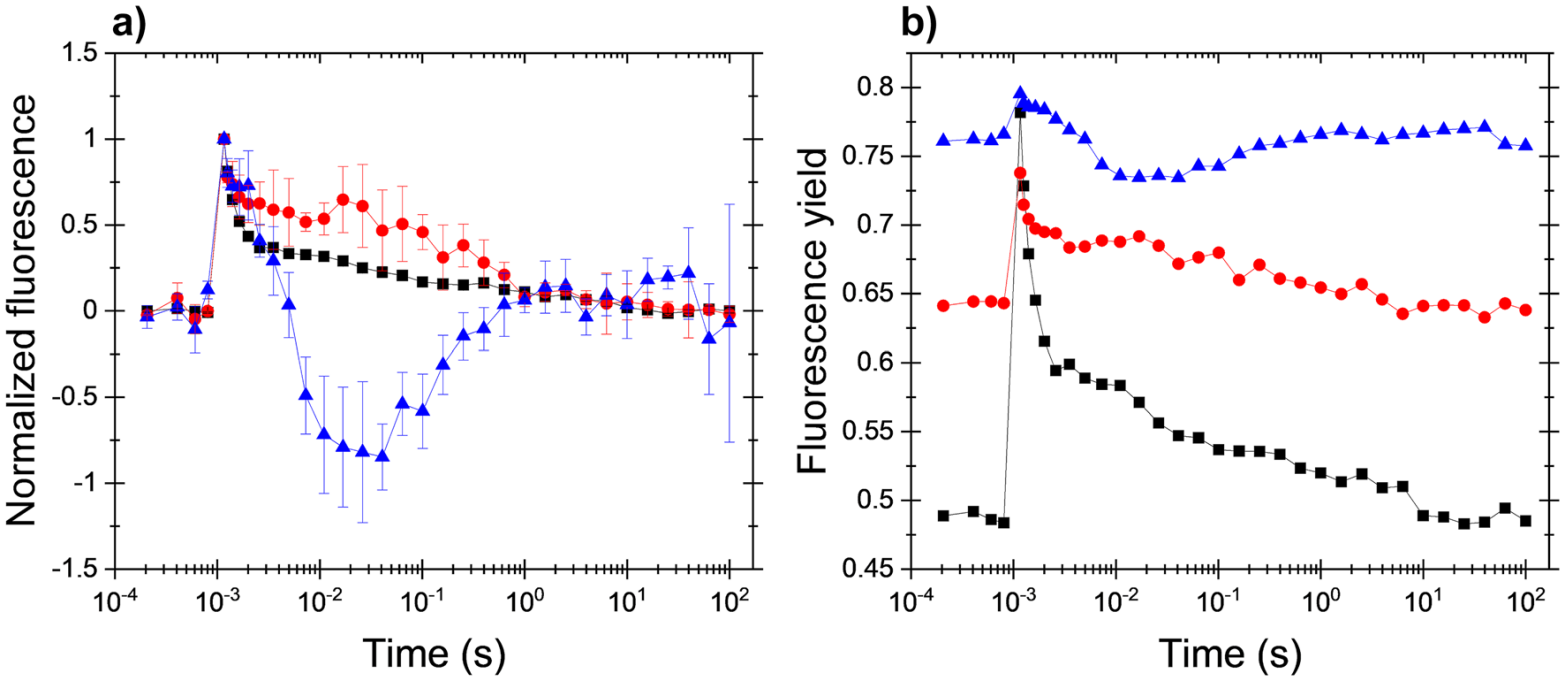
Effect of photoinhibitory treatment on the flash-induced Chl fluorescence relaxation curves in *C. reinhardtii*. Black squares: control cells (aerobic, dark-adapted cells in the presence of 1 mM lincomycin), red circles: photoinhibitory treatment (aerobic, illuminated with 2000 μmol photons m^−2^ s^−1^ white light for 15 min in the presence of 1 mM lincomycin, then dark adapted for 15 min before the measurement), blue triangles: same as photoinhibitory treatment but during dark adaptation the cell suspension was made microaerobic using the glucose oxidase reaction. **a** double normalized fluorescence traces, **b** original data

**Fig. 3:**
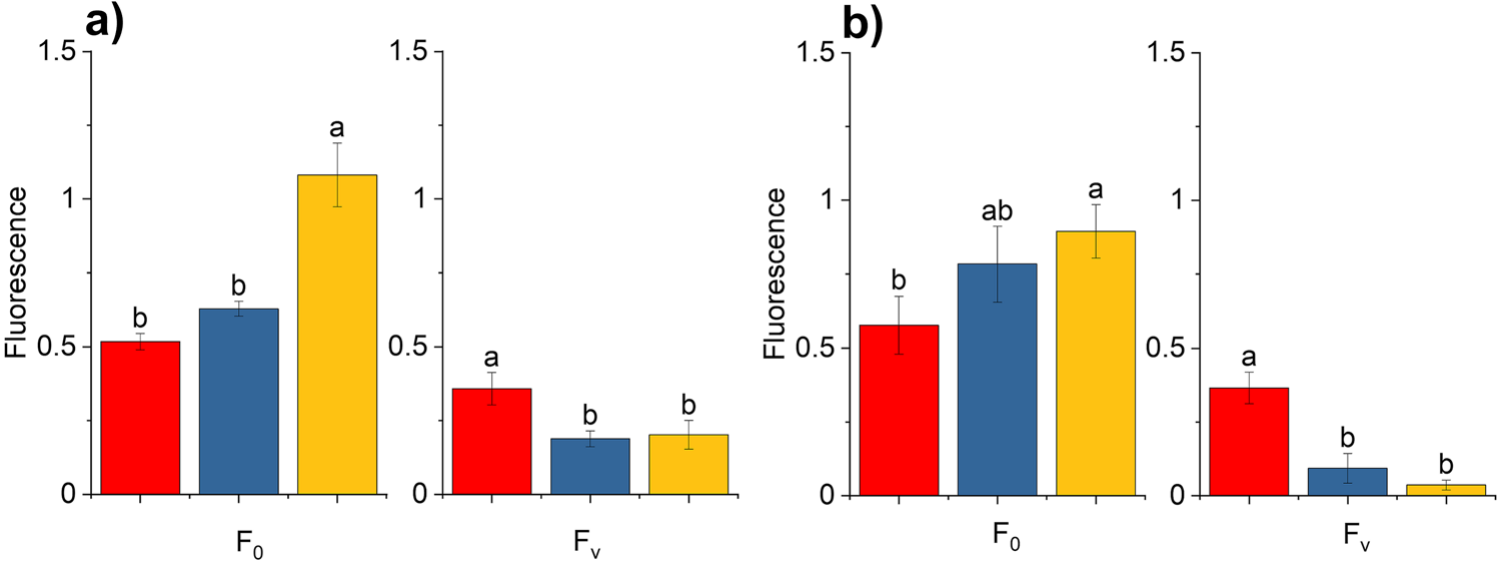
Effect of hydroxylamine and photoinhibitory treatments on fluorescence parameters. Minimum fluorescence recorded before the flash (*F*_0_) and variable fluorescence (*F*_v_), calculated as the difference of the fluorescence recorded after the single turnover flash [*F*_m(ST)_] and minimum fluorescence (*F*_0_) in the different treatments. Panel **a** Hydroxylamine treatment, panel **b** photoinhibitory treatment. Red, control aerobic (in panels **a** and **b**); blue, treated aerobic (hydroxylamine in panel a and photoinhibition in panel **b**); yellow, treated + microaerobic (hydroxylamine in panel a and photoinhibition in panel **b**). Different letters above the bars indicate statistically different means, values sharing common letters are not significantly different from one another (*p* < 0.05)

High light treatment inhibits the activity of PSII to a significantly larger extent than that of PSI (Tyystjärvi and Aro 1996; Vass 2012; Tikkanen et al. 2014) and shifts the balance of PSII:PSI electron transport rates in favor of PSI. Therefore, the effect of photoinhibitory treatment was also checked on the fluorescence relaxation kinetics. As shown in Fig. 2a the wave phenomenon could also be observed when photoinhibited cells were placed under microaerobic conditions, although the characteristics of the wave were somewhat different as compared to the wave induced by hydroxylamine + microaerobic condition (Fig. 2b shows the original fluorescence relaxation curves without normalization). Strong light in the presence of lincomycin caused a slight, statistically not significant elevation in F_0_ and a significant decrease in variable fluorescence (Fig. 3b). The fluorescence relaxation pattern after photoinhibition in microaerobic conditions exhibited an elevated middle phase, which decreased slowly in the 0.5–100 s timescale. When the photoinhibited *Chlamydomonas* cells were placed into microaerobic conditions a further increase in F_0_ was observed with a concomitant slowdown of the initial part of the fast phase of the fluorescence decay and a decrease in variable fluorescence (*F*_v_) (for statistical analysis see Fig. 3b). The fast phase was slowed down indicating the elevated reduction level of the PQ pool, similar to the condition induced with HA + microaerobic treatment. Photoinhibited *Chlamydomonas* cells in a microaerobic environment expressed a large dip in the fluorescence relaxation, after which a fluorescence increase was observed. Therefore, decrease of PSII activity by high light exposure was sufficient to induce the fluorescence wave phenomenon under microaerobic conditions. These results further corroborate the finding obtained with HA treatment that partial loss of PSII activity and concomitant elevated reduction pressure of the PQ pool (and uninhibited PSI activity) lead to the formation of the wave phenomenon of fluorescence relaxation kinetics.

### Inhibitors of cyclic electron flow indicate the involvement of the antimycin-insensitive pathway in the fluorescence wave phenomenon

**Fig. 4:**
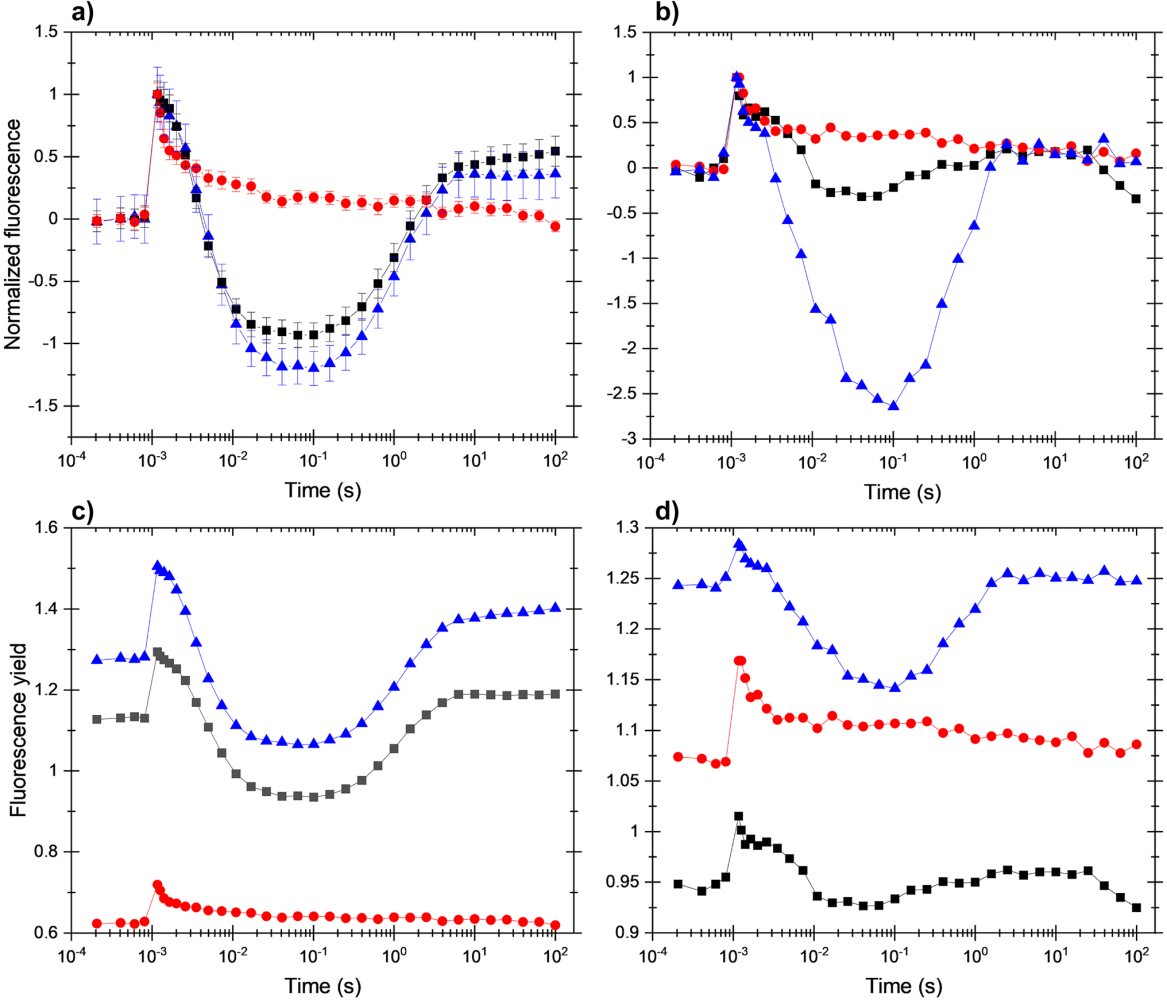
Effect of various inhibitors of cyclic electron flow on flash-induced Chl fluorescence relaxation in *C. reinhardtii* treated with HA (**a**) or photoinhibitory light (**b**) and placed to microaerobic conditions before the measurements. Black squares: samples without additional inhibitor, red circles: treated cells incubated with 400 μM polymyxin B, blue triangles: treated cells incubated with 10 μM antimycin A. Panels **a** and **b** show the double normalized fluorescence traces while panels **c** and **d** show the original traces without normalization

Previous studies indicated that the fluorescence wave phenomenon is related to an electron transport pathway mediated by the NDH-1 complex in cyanobacteria (Deák et al. 2014) and by the NDH-2 complex in *Chlamydomonas*, when the wave was induced by H_2_-producing conditions (Krishna et al. 2019). It was important to clarify whether the wave phenomenon observed here in *Chlamydomonas* under selective inhibition of PSII could also be related to an antimycin-insensitive NAD(P)H dehydrogenase (NDH-1/2) or antimycin-sensitive proton gradient regulatory (PGR5/PGRL1) pathways. To this end, various inhibitors specific to these pathways, polymyxin B and antimycin A, were applied. *C. reinhardtii* cells, which were pretreated either with HA or photoinhibitory light and then placed to microaerobic conditions. Antimycin A did not influence the fluorescence relaxation pattern significantly in the HA-treated cells (Fig. 4a), however, it caused a profound increase in the amplitude of the wave in the photoinhibited cells as compared to control cells (Fig. 4b). Polymyxin B, however, which is an inhibitor of NDH-2 dependent CEF (Krishna et al. 2019), completely abolished the formation of the wave phenomenon both in the HA-treated and photoinhibited cells (Fig. 4a and b, respectively, original fluorescence relaxation curves without normalization are shown in Fig. 4c, d, respectively).

## Discussion

Flash-induced Chl fluorescence relaxation reflects sequential electron transport at the acceptor side of PSII due to reoxidation of Q_A_^−^ by Q_B_ and PQ molecules in the PQ pool and it also gives information about the charge recombination processes between the oxidized S_2_ state of the water oxidizing complex and Q_A_Q_B_^−^ (Crofts and Wraight 1983; Robinson and Crofts 1983; Vass et al. , 1992, 1999). A special case of fluorescence relaxation is the appearance of the so-called wave phenomenon, which is a transient reduction-reoxidation and re-reduction of Q_A_ due to changes in the redox state of the PQ pool, which is initiated by the imbalance of electron flow mediated by PSII and PSI. This effect was first observed and characterized in the cyanobacterium *Synechocystis* PCC 6803 under microaerobic conditions (Deák et al. 2014). Other observations however showed that microaerobic conditions alone are neither necessary nor sufficient for the appearance of the fluorescence wave. On the one hand the fluorescence wave was observed under aerobic conditions in the cyanobacteria *Thermosynechococcus elongatus* (Deák et al. 2014), and site-directed mutants *Thermosynechococcus vulcanus* in which the amino acid residues that are responsible for the binding of the PsbV to the PSII core were modified (Xiao et al. 2020), as well as in the red morphotype of the microalga, *Acetabularia acetabulum* (Havurinne and Tyystjärvi 2020). On the other hand the fluorescence wave phenomenon was not observed in *Chlamydomonas* under microaerobic conditions (Volgusheva et al. 2013, 2016; Krishna et al. 2019) or was present only in small extent (Fig. 1a in the present study).

### The fluorescence wave phenomenon requires both an unbalanced ratio of PSII and PSI activities and highly reduced PQ pool

Cyanobacteria, which show the fluorescence wave, have typically low PSII:PSI ratio (1:3–5 (Aizawa et al. 1992)). In addition, in *Chlamydomonas* the fluorescence wave was previously observed only under H_2_-producing conditions in sulphur-deprived cells (Volgusheva et al. 2013, 2016; Krishna et al. 2019) when microaerobic conditions were accompanied with partly inhibited PSII activity. These observations prompted us to explore the hypothesis that the ratio of PSII and PSI electron transport activity could be a critical factor in the appearance of the fluorescence wave. To clarify this point treatments were applied to *Chlamydomonas* cells which induce partial inhibition of PSII activity, but do not affect PSI activity.

One of the applied inhibitory treatments was the addition of HA, which impairs the donor side of PSII by releasing the Mn cluster (Cheniae and Martin 1971; Vass et al. 1999; Tóth et al. 2007), but does not affect PSI. The other treatment was high light exposure which decreases the activity of PSII relative to that of PSI (Tyystjärvi and Aro 1996; Vass 2012; Tikkanen et al. 2014). The data obtained from these experiments demonstrated that the fluorescence wave can be induced in *Chlamydomonas* by selective inhibition of PSII activity and confirm our hypothesis that a prerequisite for the appearance of the wave phenomenon is a significant imbalance of PSII and PSI activities in favor of PSI, which makes possible a rapid transient oxidation of the PQ pool after the flash. This PSI-driven transient PQ pool oxidation is reflected by the dip of the fluorescence transient.

The imbalance of PSII and PSI activities arises from a low PSII:PSI ratio in most cyanobacteria, such as *Synechocystis* PCC 6803, or *Thermosynechococcus elongatus*. The lack of the fluorescence wave in *Acaryochloris marina* (Deák et al. 2014) is possibly related to the higher PSII:PSI ratio in *Acaryochloris* (Mimuro et al. 2004; Itoh et al. 2007) as compared to *Synechocystis*, resulting in a well-balanced electron injection to and electron withdrawal from the PQ pool, by PSII and PSI, respectively, which prevents the transient reoxidation of PQ pool, therefore also preventing the formation of the wave.

Earlier studies have established that another critical condition for the appearance of the fluorescence wave is the high reduction level of the PQ pool (Deák et al. 2014). This makes possible the transmittance of transient redox changes, which are induced by the imbalanced electron injection to and withdrawal from of the PQ pool by PSII and PSI, respectively back to the level of Q_A_, and therefore allowing the use of Chl fluorescence yield changes to follow these redox changes. The high reduction level of the PQ pool can be established by microaerobic conditions, which prevent PQ oxidation, and several observations of the fluorescence wave was in microaerobicity. However, in some cases the fluorescence wave was observed under aerobic conditions, e.g. in the cyanobacterium *Thermosynechococcus elongatus* (Deák et al. 2014), and in the in the red morphotype of microalga *Acetabularia acetabulum* (Havurinne and Tyystjärvi 2020). In *Thermosynechococcus elongatus* the PQ pool can be sufficiently reduced even under aerobic conditions to allow the wave formation via respiratory electron donation as shown by the sustained level of post-illumination Chl fluorescence (Deák et al. 2014). Similar effect might occur in *Acetabularia acetabulum* via strong non-photochemical reduction of the PQ pool in darkness possibly via respiratory electron donation (Havurinne and Tyystjärvi 2020).

### The fluorescence wave phenomenon reflects NDH-mediated electron transport pathways

The fluorescence wave in *Synechocystis* has been clearly assigned to the activity of the NDH-1 complex (Deák et al. 2014). In *Chlamydomonas* the wave phenomenon was observed under H_2_-evolving conditions, which was found to be related to the activity of the NDH-2 complex. This was proven by the absence of the wave in the presence of the NDH-2 inhibitor polymyxin B and in the *Δnda2* mutant, which lacks NDA2 (Krishna et al. 2019). Our data provide support to the previous finding of Krishna et al. (Krishna et al. 2019) since the wave phenomenon could be blocked by the NDA2 inhibitor polymyxin B, but not with antimycin A in the case of both HA-treated and photoinhibited cells. In *C. reinhardtii*, CEF was also found to be insensitive to antimycin A (Iwai et al. 2010), which is in agreement with our study in that the wave phenomenon could not be abolished by antimycin A, however this response is likely strain-dependent (Antal et al. 2013). Interestingly, the amplitude of the fluorescence dip was even enhanced in the presence of antimycin A, especially in photoinhibited cells (Fig. 4b). This effect might be related to the ability of antimycin A to accelerate the reduction of plastoquinone in the dark (Antal et al. 2013). On the other hand, the elevated H_2_ production in the presence of antimycin A (Krishna et al. 2019) and in the *pgr5* and *pgrl1* mutants (Nagy et al. 2021) indicate the existence of an antimycin-sensitive pathway, which competes for the electrons that are required for H_2_ production in *C. reinhardtii* (see also Tolleter et al. 2011; Godaux et al. 2015).

Even though the fluorescence wave is eliminated in the absence of the NDH-1 complex in *Synechocystis* (Deák et al. 2014), and by polymyxin B in *Chlamydomonas* as shown earlier (Krishna et al. 2019) and here, it is unlikely that the wave phenomenon would reflect an actual flash-induced cycling of electrons from the acceptor side of PSI back to the PQ pool. This idea is based on the slow rise, several seconds (Figs. 1, 2, 4), of the fluorescence intensity during the bump phase following the dip, which is much slower than ms timescale re-reduction of P700^+^ via cyclic electron flow (e.g. Alric 2010). The slow re-reduction of PQ pool in microaerobic condition is in agreement with previous observations in anoxic, dark-adapted *Chlamydomonas* cells, and the capacity of CEF was not found to be affected by PGRL1 (Nawrocki et al. 2019). In *Chlamydomonas*, the plastid terminal oxidase PTOX (especially PTOX2) oxidizes plastoquinone pool with a rate of approx. 5 e^−^ s^−1^, and considering that the rate constants of reduction and oxidation of PQ pool are of similar magnitude, the absence of PTOX2 leads to PQ reduction probably with a similar rate (Houille-Vernes et al. 2011). We suggest that refilling of the PQ pool following its transient oxidation by PSI after the flash is due to an electron flow from stromal components to the PQ pool, which is mediated by NDH-1 in cyanobacteria and NDH-2 in *Chlamydomonas* and possibly in other microalgae.

## Concluding remarks

Our data confirm previous findings that the appearance of a wave in the relaxation of flash-induced Chl fluorescence yield is a general phenomenon that occurs not only in cyanobacteria (Deák et al. 2014), but also in *Chlamydomonas* (Volgusheva et al. 2013, 2016; Krishna et al. 2019) and other microalgae (Havurinne and Tyystjärvi 2020) under appropriate conditions.

The data presented here also demonstrate that the significantly imbalanced PSII and PSI activity in favor of PSI is an equally important condition for the appearance of the fluorescence wave as the highly reduced state of the PQ pool. The characteristic appearance of the wave phenomenon is therefore a potentially useful physiological marker of stress conditions that modulate the PSII:PSI ratio or impact PSII activity relative to PSI activity, as well as affect the redox state of the PQ pool. Whether this wave phenomenon shows species-specific characteristics in microalgae remains to be investigated.

## Data Availability

The datasets generated during and/or analyzed during the current study are available from the corresponding authors on reasonable request.

## Abbreviations

CEF: Cyclic electron flow
Chl: Chlorophyll
HA: Hydroxylamine
NDH: NAD(P)H dehydrogenase
PGR5: Proton gradient regulation 5
PGRL1: PGR5-like photosynthetic phenotype 1
PQ: Plastoquinone
PSII: Photosystem II
PSI: Photosystem I
